# Changes in OCT4 expression play a crucial role in the lineage specification and proliferation of preimplantation porcine blastocysts

**DOI:** 10.1111/cpr.13313

**Published:** 2022-07-26

**Authors:** Mingyun Lee, Jong‐Nam Oh, Gyung Cheol Choe, Seung‐Hun Kim, Kwang‐Hwan Choi, Dong‐Kyung Lee, Jinsol Jeong, Chang‐Kyu Lee

**Affiliations:** ^1^ Department of Agricultural Biotechnology, Animal Biotechnology Major, and Research Institute of Agriculture and Life Sciences Seoul National University Seoul South Korea; ^2^ Institute of Green Bio Science and Technology Seoul National University Pyeongchang South Korea; ^3^ Present address: Research and Development Center Space F Corporation Hwasung South Korea

## Abstract

**Objectives:**

Curiosity about the role of OCT4, a core transcription factor that maintains inner cell mass (ICM) formation during preimplantation embryogenesis and the pluripotent state in embryonic development, has long been an issue. OCT4 has a species‐specific expression pattern in mammalian preimplantation embryogenesis and is known to play an essential role in ICM formation. However, there is a need to study new roles for OCT4‐related pluripotency networks and second‐cell fate decisions.

**Materials and Methods:**

To determine the functions of OCT4 in lineage specification and embryo proliferation, loss‐ and gain‐of–function studies were performed on porcine parthenotes using microinjection. Then, we performed immunocytochemistry and quantitative real‐time polymerase chain reaction (PCR) to examine the association of OCT4 with other lineage markers and its effect on downstream genes.

**Results:**

In OCT4‐targeted late blastocysts, SOX2, NANOG, and SOX17 positive cells were decreased, and the total cell number of blastocysts was also decreased. According to real‐time PCR analysis, NANOG, SOX17, and CDK4 were decreased in OCT4‐targeted blastocysts, but trophoblast‐related genes were increased. In OCT4‐overexpressing blastocysts, SOX2 and NANOG positive cells increased, while SOX17 positive cells decreased, and while total cell number of blastocysts increased. As a result of real‐time PCR analysis, the expression of SOX2, NANOG, and CDK4 was increased, but the expression of SOX17 was decreased.

**Conclusion:**

Taken together, our results demonstrated that OCT4 leads pluripotency in porcine blastocysts and also plays an important role in ICM formation, secondary cell fate decision, and cell proliferation.

## Graphical abstract

We analyzed the phenotypes of OCT4 knockout and OCT4 overexpressing preimplantation porcine embryos to understand the function of OCT4, one of the core transcription factors. In the OCT4 targeted embryos, intact inner cell mass (ICM) was not formed, and the expression of pluripotency‐related genes and proliferation‐related genes was decreased. In OCT4 overexpression embryos, the expression of the primitive endoderm marker SOX17 was decreased and the expression of epiblast marker genes increased. These results suggest that OCT4 is involved in both cell fate decisions in porcine preimplantation embryos and also affects cell proliferation.

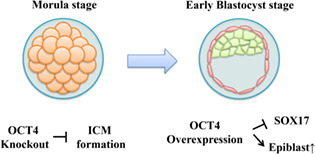

## 1 INTRODUCTION

Mammalian embryos undergo two cell fate decisions during preimplantation embryogenesis.^1^ The first decision is divided into an inner cell mass and trophectoderm,^2^ and the second decision segregates ICM into epiblast and primitive endoderm.^3^, ^4^ Numerous genes and mechanisms are involved in this lineage specification, and OCT4 has been heavily studied due to it's importance. OCT4 is one of the core transcription factors of pluripotency, has a DNA‐binding domain, and controls expression by binding to a consensus sequence.^5^ In addition, OCT4 plays a key role in maintaining and re‐establishing pluripotency; is specifically highly expressed in pluripotent cells, such as embryos, embryonic stem cells, germ cells, and iPS cells; and is rapidly reduced when differentiated.^6^, ^7^, ^8^ The absence of OCT4 expression leads to the differentiation of pluripotent cells and the non‐ICM formation of embryos,^9^, ^10^, ^11^ but overexpression of OCT4 leads to differentiation.^12^ In addition, the downregulation of OCT4 expression by siRNA reduces the binding to each target of Smad1 and STAT3, to the components of bone morphogenetic protein (BMP), and to leukaemia inhibitory factor (LIF) signalling.^13^ In terms of gain of function, it was found that the overexpression of OCT4 in mouse embryos arrests developmental processes, suggesting that the strict control of OCT4 levels is essential.^14^ Therefore, OCT4 is considered to be a master regulator that maintains a pluripotent state in embryos and embryonic stem cells.^5^, ^15^

Although OCT4 plays an important role in early embryogenesis, it has species‐specific characteristics.^16^ In the blastocyst of mice, OCT4 is expressed only in the ICM, whereas in pigs and cattle, OCT4 is expressed not only in the ICM but also in the trophectoderm.^17^ Furthermore, it has been shown that the pattern of OCT4 expression during pig embryogenesis is more common in humans than in mice.^10^, ^11^, ^18^ In addition, as pig embryonic stem cells were established,^19^ the proximal enhancer and distal enhancer, which are upstream regulatory elements of OCT4, were analyzed.^20^, ^21^, ^22^ In the case of mice, the proximal enhancer works in epiblast and epiblast stem cells, and the distal enhancer works in ICM,^23^ whereas in pigs, both enhancers work in ICM and embryonic stem cells, which shows species‐specific characteristics even in the use of enhancers (accepted in *Stem Cells International*).^24^ Therefore, species‐specific OCT4 studies are needed. The development of genetic manipulation technology has facilitated the generation of genetically modified embryos, accelerating both preimplantation embryo lineage specification and pluripotency network research.^25^, ^26^, ^27^ Many studies have attempted to elucidate the role of OCT4, SOX2, and NANOG, the core transcription factors of pluripotency in various species, in the preimplantation embryo. The disruption of OCT4 expression in mammalian embryos prevents ICM formation and the maintenance of pluripotency.^28^, ^29^, ^30^ SOX2 is known to play an important role in the formation of ICM or intact blastocysts.^18^, ^31^ In NANOG knockout embryos, epiblast formation is disturbed, and the ICM differentiates into the primitive endoderm.^32^, ^33^ Conversely, the roles in the lineage specification of CDX2 and DAB2, known as trophoblast markers, were analyzed.^10^, ^34^, ^35^, ^36^, ^37^ Several more advanced studies have attempted to elucidate the expression relationship of OCT4 and SOX2 in ICM formation by analyzing the upstream mechanisms of core transcription factors. Recently, it was revealed in mouse embryos that the asymmetric formation of CARM1 and paraspeckles is an upstream mechanism for zygotic ICM‐specific gene expression regulation.^38^, ^39^ In addition, the ICM formation mechanism system was analyzed by quantifying the binding of OCT4 and SOX2^40^ to genomic DNA. However, despite these attempts, more data are needed on the regulatory mechanisms between the two genes and the networks of other pluripotent genes during early embryogenesis. Furthermore, varied roles for OCT4 in porcine embryos need to be demonstrated by knockout and overexpression assays. Therefore, in this study, we investigated the overall role of OCT4 and its relationship with other lineage marker genes during the preimplantation development of ‐ porcine embryos. First, we analyzed the correlation with the expression patterns of lineage markers during blastocyst formation. And, to investigate the role of OCT4, the verified sgRNA and Cas9 mRNA were injected into the porcine embryo and then analyzed. Next, we induced overexpression by injecting exogenous porcine *OCT4* mRNA into embryos and analyzed the changes in lineage marker genes at the blastocyst stage. This trial revealed a role for OCT4 in lineage specification and proliferation during preimplantation embryo development and will help to elucidate the network between pluripotency‐related genes.

## 2 RESULTS

### 2.1 Expression analysis of cell fate markers in porcine preimplantation embryos

**FIGURE 1:**
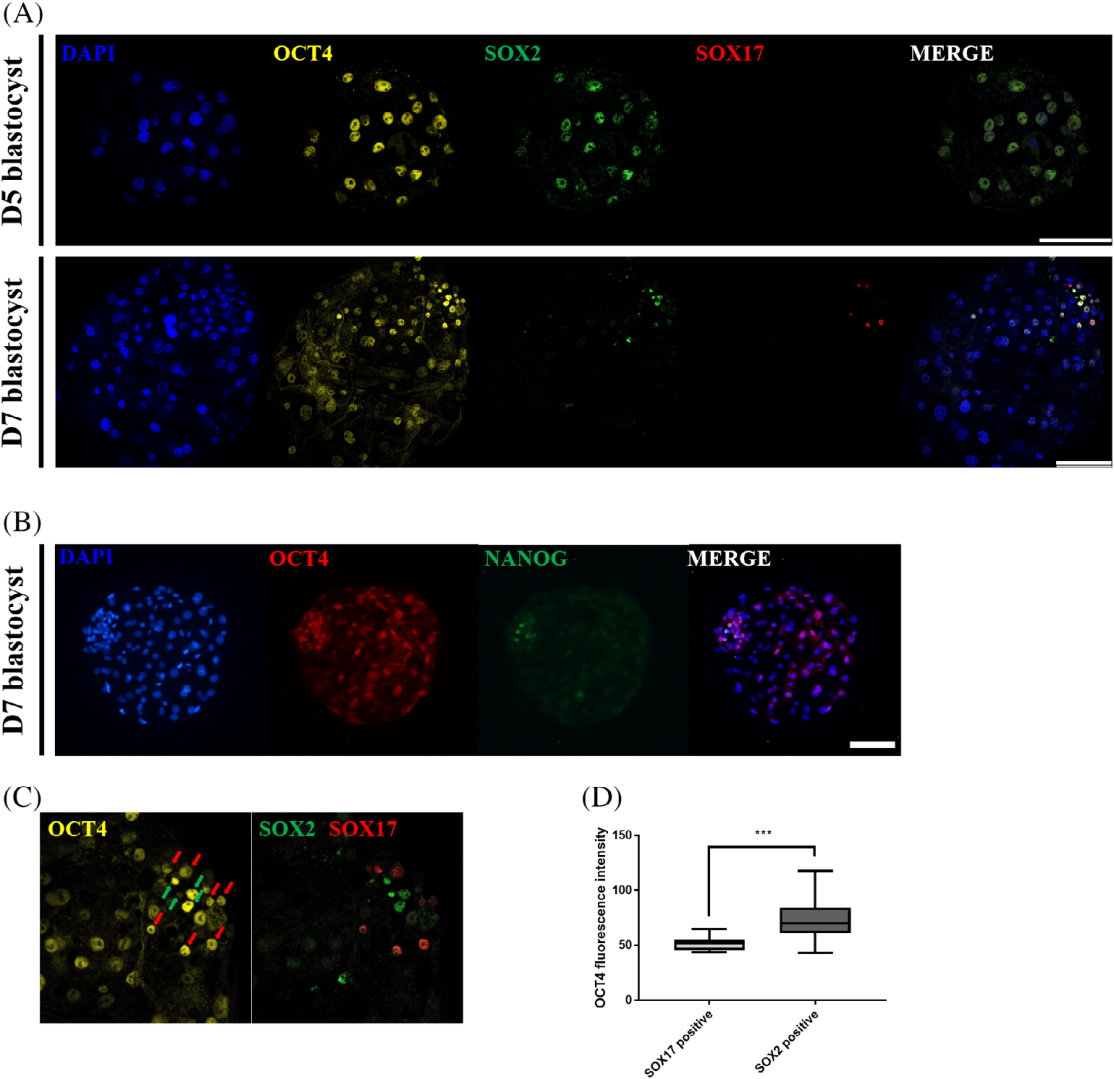
The immunolocalization of OCT4, SOX2, SOX17, and NANOG in porcine preimplantation blastocysts. (A) Expression and localization of lineage marker genes (OCT4, SOX2, and SOX17) in porcine blastocysts. Nuclei were stained with DAPI using yellow for OCT4, green for SOX2, and red for SOX17. The size marker corresponds to 100 μm. (B) Expression pattern of OCT4 and NANOG in porcine D7 blastocysts. Nuclei were stained with DAPI, OCT4 with red, and NANOG with green. The size marker corresponds to 100 μm. (C) Relationship between the fluorescence intensity of OCT4 and the expression of SOX2 and SOX17. Green arrows indicate SOX2‐positive cells, and red arrows indicate SOX17‐positive cells. (D) Comparison of OCT4 fluorescence intensity between the SOX2‐ and SOX17‐positive cell group. Sample size of OCT4/SOX2/SOX17 was *n* = 10. Error bars represent the mean SEM, *significant differences (***p < 0.001)

OCT4, which is important for maintaining pluripotency, shows a distinct expression pattern in pig early embryogenesis compared to that of mice. Therefore, in porcine preimplantation embryos, spatiotemporal patterns were analyzed to examine the expression patterns and relationships of lineage markers and determine what characteristics these distinct differences elicit. To reduce sperm‐derived variables, embryos by parthenogenetic activation were used. In particular, triple staining analysis was performed on three genes (OCT4, SOX2, and SOX17) to analyze the expression of several genes in one blastocyst. In the D5 blastocyst, OCT4 was expressed together with SOX2, but SOX17 was not expressed. In the D7 blastocyst, OCT4 was expressed in both the ICM and TE, but it showed higher staining intensity in the ICM. SOX2 and SOX17 were expressed in different subpopulations in the D7 blastocyst ICM (Figure 1A), and NANOG was expressed in only one subpopulation (Figure 1B). The relationship between these cell fate markers could be found, and it was found that SOX2‐ and SOX17‐positive cells with a high staining intensity not only constituted their populations but were also related to the staining intensity of OCT4. Most of the SOX2‐positive cells had high OCT4 staining intensity, whereas SOX17‐positive cells had significantly low OCT4 staining intensity (Figure 1C, D). These results show that OCT4 is linked to the cell fate marker network. Therefore, we studied the role of OCT4 during porcine preimplantation lineage specification by means of its gain and loss of function.

### 2.2 Effects of OCT4 knockout during porcine preimplantation embryo development

**FIGURE 2:**
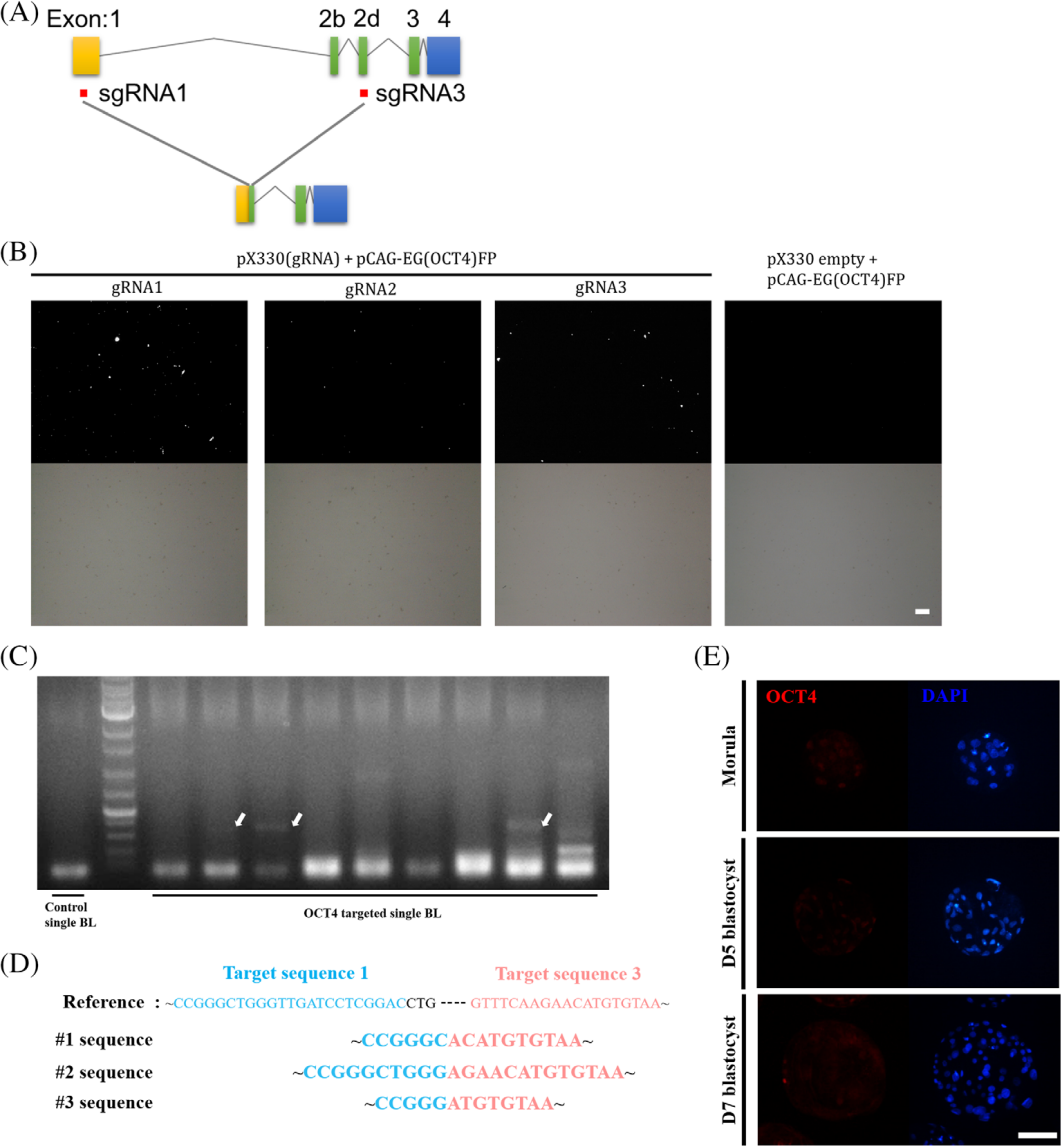
gRNA validation for OCT4 editing with the CRISPR/Cas9 system. (A) Schematic representation of the porcine OCT4 locus and the targeting sites of gRNA1 and gRNA3. (B) The cleavage efficiency of pX330, which contained the gRNA 1–3 sequences, in the porcine OCT4 region of the pCAG‐EGxxFP vector. The size marker corresponds to 50 μm. (C) Agarose visualization of genotyped OCT4‐targeted D7 blastocysts. The white arrow indicates the shortened OCT4 genomic DNA by dual targets in A. (D) The deletion of the Sanger sequencing result of the white arrow D7 blastocysts in C. (E) Immunofluorescence analysis for OCT4 (red) and DAPI nuclear staining in OCT4‐targeted morula, D5 blastocyst, and D7 blastocyst stages. The size marker corresponds to 100 μm.

**FIGURE 3:**
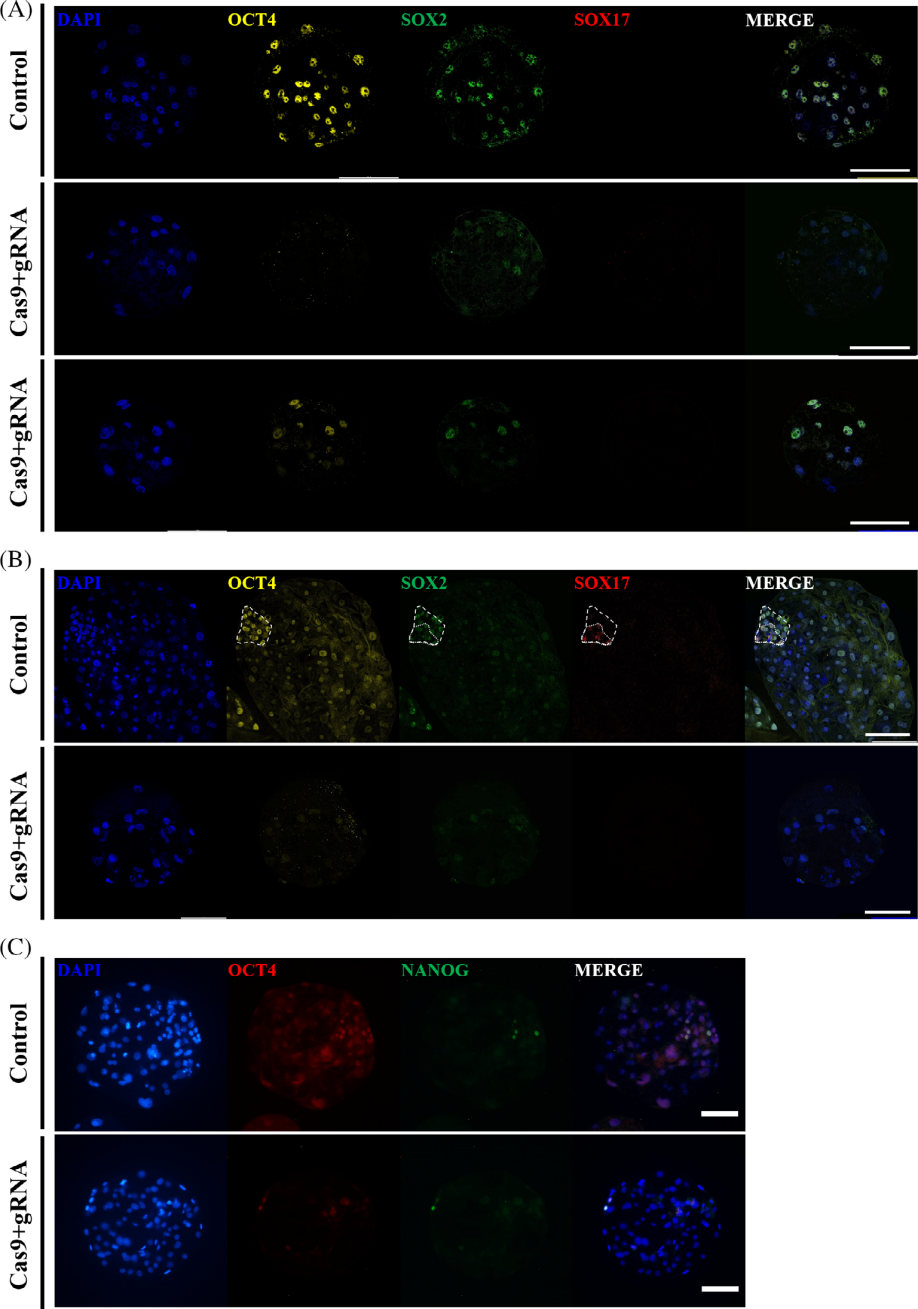
The knockout of OCT4 in embryos prevents the expression of ICM‐specific genes. (A) Expression of SOX2 and SOX17 in control D5 blastocysts and OCT4‐targeted D5 blastocysts. Nuclei were stained with DAPI using yellow for OCT4, green for SOX2, and red for SOX17. Sample size was *n* = 10. The size marker corresponds to 100 μm. (B) Expression of SOX2 and SOX17 in control D7 blastocysts and OCT4‐targeted D7 blastocysts. Thick dotted line: ICM, thin dotted line: primitive endoderm. Sample size was *n* = 10. The size marker corresponds to 100 μm. (C) Expression of NANOG in control D7 blastocysts OCT4‐targeted D7 blastocysts. Nuclei were stained with DAPI, using red for OCT4 and green for NANOG. Sample size was *n* = 10. The size marker corresponds to 100 μm.

To analyze the role of OCT4 in porcine preimplantation embryos, a knockout assay using CRISPR/Cas9 was performed. We selected target sequences are located in the exon1 and exon2d of porcine OCT4, and the distance between the two sequences is 4385 bp (Figure 2A). The two types of high‐efficiency sgRNA were selected from out of three porcine OCT4 target sequences using the pCAG‐EGxxFP system as described in a previous study.^18^ In the negative control, GFP was not expressed, and in the treatment group, GFP was highly expressed in the gRNA1 and gRNA3 groups. (Figure 2B). The selected gRNAs were microinjected with Cas9 mRNA into parthenote, and genomic DNA deletion was confirmed at the D7 blastocyst stage (Figure 2C). The sequence between gRNA1 and gRNA3 was deleted by sequencing a fragment of approximately 300 bp in size among deleted genomic OCT4 of several lengths (Figure 2D). In addition, OCT4 was not expressed in morula, a D5 blastocyst, and a D7 blastocyst were injected with gRNAs and Cas9 mRNA, and CRISPR/Cas9 was effectively introduced and worked correctly (Figure 2E). To investigate the role of OCT4 in porcine preimplantation embryos, immunofluorescence analysis was performed. In the D5 blastocyst control group, OCT4 and SOX2 were expressed and overlapped in most cells, but SOX17 was not expressed (Figure 3A). On the other hand, OCT4‐targeted D5 blastocysts did not express OCT4, and the expression of SOX2 was reduced. In particular, in blastocysts with an OCT4 knockout mosaic pattern, OCT4 was expressed only in some cells, and SOX2 was expressed only in OCT4‐expressing cells. In the D7 OCT‐targeted blastocyst, the ICM was not formed, and SOX2, an epiblast marker, and SOX17, a primitive endoderm marker, were not expressed (Figure 3B). NANOG was expressed in some ICM cells in the control group but was not expressed in the OCT4‐targeted group or was expressed only in some cells expressing OCT4 (Figure 3C). Furthermore, the total cell number of OCT4‐targeted D7 blastocysts was also significantly decreased compared to that of the control group, and since ICM was not formed, SOX2‐, NANOG‐, and SOX17‐positive cells were also significantly decreased (Table 1).

**TABLE 1:** The number of tatal cells and pluripotent marker positive cells in OCT4 targeted blastocysts

Group	No. blastocyst (*n* = 3)	Cells in blastocysts			
		Total cell number	SOX2 positive cells	NANOG positive cells	SOX17 positive cells
Cas9 mRNA.	30	139 ± 5.3^a^	10.4 ± 0.7^a^	6.8 ± 1.4^a^	6.45 ± 0.6^a^
Cas9 mRNA+ sgRNA	30	55.1 ± 4.3^b^	0.5 ± 0.2^b^	0.8 ± 0.4^b^	0.6 ± 0.2^b^

*Notes*: The number of cells was counted in the late blastocyst. Data presented as mean ± SE. Values with different letters (a and b) are significantly different (*p* < 0.05).

**FIGURE 4:**
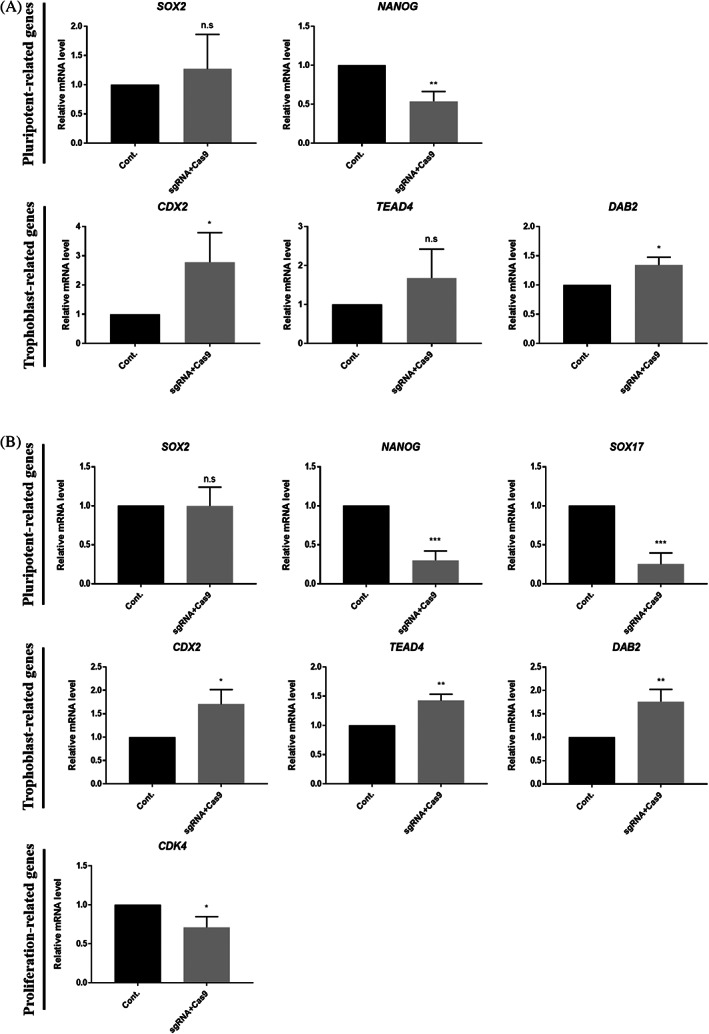
Lineage marker genes and proliferation gene expression patterns in OCT4‐targeted blastocysts. (A) Transcription levels of pluripotency and trophoblast‐related genes shown for control and OCT4‐targeted D5 blastocysts. Sample size was *n* = 30. Each group had three replicates. Error bars represent the mean SEM *corresponds to significant differences (*p < 0.05, **p < 0.01, ***p < 0.001). (B) Transcription levels of pluripotency‐, trophoblast‐ and proliferation‐related genes shown for control and OCT4‐targeted D7 blastocysts. Sample size was *n* = 30. Each group had three replicates. Error bars represent the mean SEM *corresponds to significant differences (*p < 0.05, **p < 0.01, ***p < 0.001).

Quantitative polymerase chain reaction (qPCR) analysis showed that in the D5 blastocyst OCT4‐targeted group, there was no significant difference in *SOX2* expression level compared to that of the control group, but the expression of *NANOG* was significantly reduced (Figure 4A). In the case of trophoblast‐related genes, the expression of *CDX2* and *DAB2*, except for *TEAD4*, was increased in D7 blastocysts, except for *SOX2*; the expression of *NANOG* and *SOX17* was significantly decreased; and the expression of trophoblast‐related genes, such as *CDX2*, *TEAD4*, and *DAB2*, was significantly increased (Figure 4B). Furthermore, the expression of *CDK4*, a proliferation‐related gene, was decreased. Taken together, OCT4 knockout in early‐stage porcine embryos disrupts lineage specification and developmental proliferation.

### 2.3 Effects of OCT4 overexpression on porcine embryo lineage specification

**FIGURE 5:**
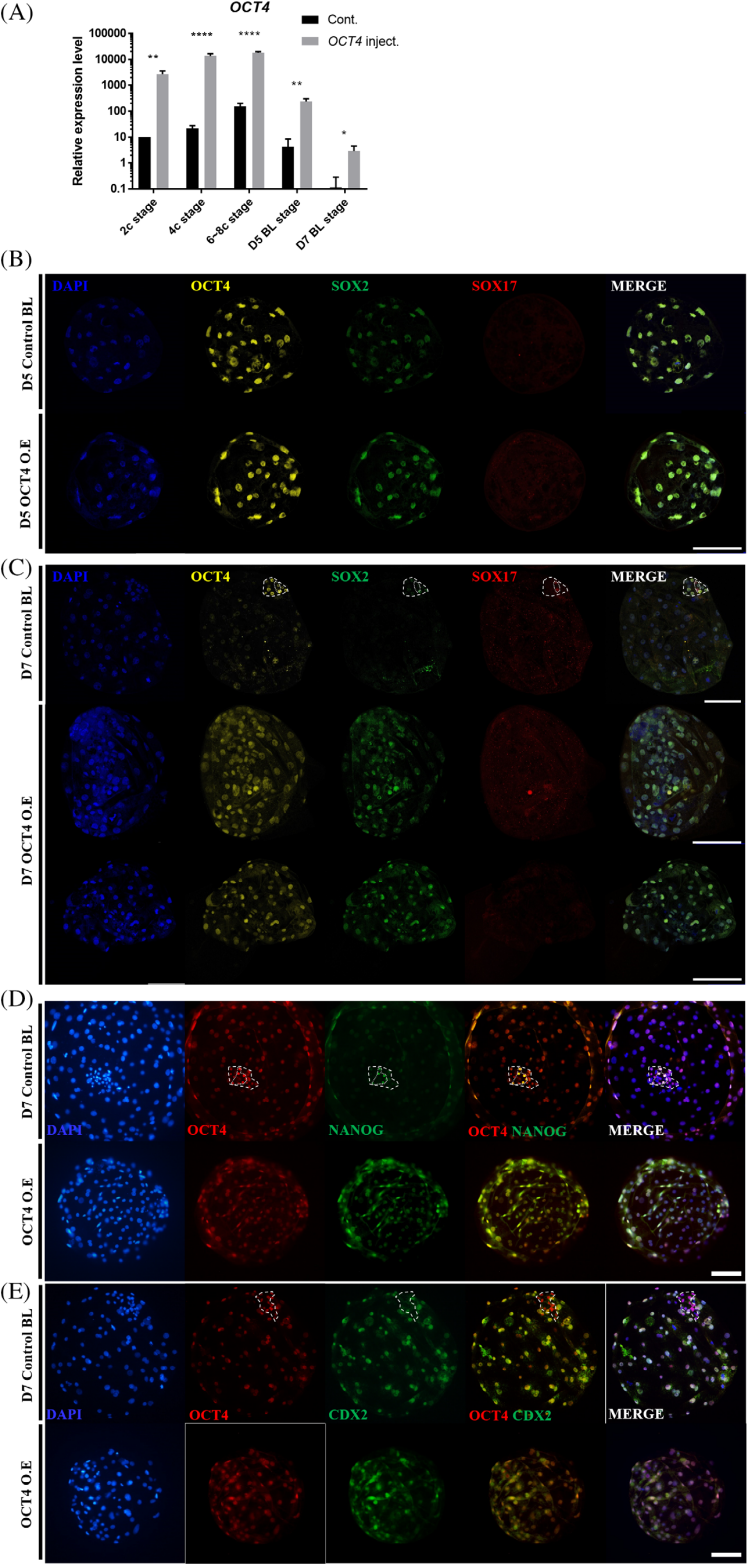
The effects of OCT4 overexpression on lineage marker genes in porcine blastocysts. (A) The transcription level of *OCT4* in embryos injected with *OCT4* mRNA during preimplantation embryogenesis. The sample size was *n* = 10 for each stage, and each group had three replicates. Error bars represent the mean SEM *corresponds to significant differences (*p < 0.05, **p < 0.01, ***p < 0.001, ****p < 0.0001). (B) and (C) Immunofluorescence analysis for DAPI and OCT4 (yellow), SOX2 (green), and SOX17 (red) staining in OCT4‐overexpressing D5 and D7 blastocysts. Sample size of OCT4/SOX2/SOX17 was *n* = 10 for each stage. Thick dotted line: ICM, thin dotted line: primitive endoderm. The size marker corresponds to 100 μm. (D) Immunofluorescence analysis of DAPI, OCT4 (red), and NANOG (green) staining in OCT4‐overexpressing D7 blastocysts. Thick dotted line: ICM, thin dotted line: epiblast. The sample size was *n* = 10. The size marker corresponds to 100 μm. (E) Immunofluorescence analysis for DAPI and OCT4 (red) and CDX2 (green) staining in OCT4‐overexpressing D7 blastocysts. Thick dotted line: ICM. The sample size was *n* = 10. The size marker corresponds to 100 μm.

Next, we conducted a cytoplasmic injection assay of *OCT4* mRNA at the one‐cell stage to investigate the role of OCT4 overexpression in the pig preimplantation developmental process. Quantitative PCR assays were performed during development to verify the injection and maintenance of *OCT4* mRNA in embryos (Figure 5A). In all stages of injected embryos, OCT4 mRNA was increased more than in control embryos, and the difference was maintained until the occurrence of the D7 blastocyst. As a result of the immunocytochemistry assay, but there was no significant difference in D5 blastocyst stage (Figure 5B). On the other hand, in OCT4‐overexpressing D7 blastocysts, the staining intensity of OCT4 remained high throughout the blastocyst, so the difference in OCT4 expression between ICM and TE was not clear (Figure 5C). In addition, the expression of SOX2 was not limited to ICM and was expressed in most cells of the blastocyst. Interestingly, the number of SOX17‐positive cells was greatly reduced compared to that in the control group (Table 2). Therefore, it can be assumed that differentiation into primitive endoderm is interrupted in OCT4 overexpressing D7 blastocysts. NANOG was expressed in most blastocyst cells of OCT4‐overexpressing D7 blastocyst, which was different from the control, in which NANOG was expressed only in a part of the ICM (Figure 5D). CDX2 was expressed in most cells except for the ICM in the control group, but there was no significant difference in OCT4‐overexpressing blastocysts. Furthermore, the total cell number of OCT4 mRNA injected D7 blastocysts was significantly higher than that of the control group (Table 2). The above results confirmed that OCT4 affects first and second lineage specifications and cell proliferation in preimplantation embryos and leads to the expression of pluripotency genes.

**TABLE 2:** The number of tatal cells and pluripotent marker positive cells in OCT4 overexpressed blastocysts

Group	No. blastocyst (*n* = 3)	Cells in blastocysts			
		Total cell number	SOX2 positive cells	NANOG positive cells	SOX17 positive cells
Control	30	141 ± 4.6^a^	10.7 ± 0.7^a^	6.6 ± 0.5^a^	5.5 ± 0.5^a^
OCT4 mRNA injected	30	160.8 ± 6.0^b^	105.3 ± 10.7^b^	90.2 ± 7.8^b^	0.7 ± 0.3^b^

*Notes*: The number of cells was counted in the late blastocyst. Data presented as mean ± SE. Values with different letters (a and b) are significantly different (*p* < 0.05).

**FIGURE 6:**
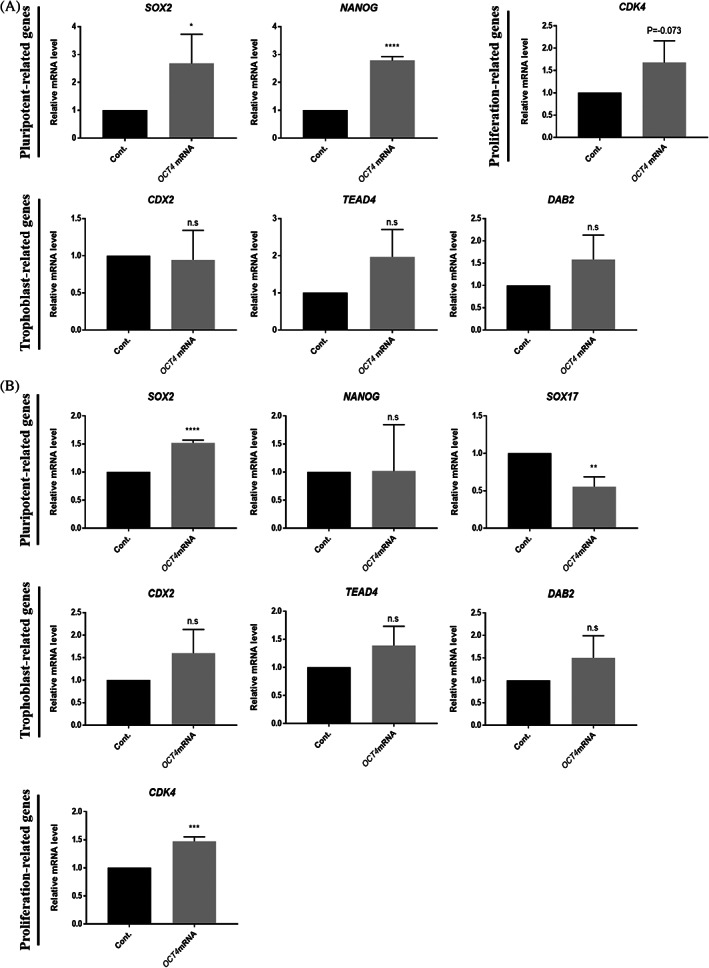
Lineage marker genes and proliferation gene expression patterns in OCT4‐overexpressing blastocysts. (A) and (B) Transcription levels of pluripotency‐, trophoblast‐ and proliferation‐related genes shown for control and OCT4‐overexpressing D5 and D7 blastocysts. Each group had three replicates. Sample size was *n* = 30. Error bars represent the mean SEM*corresponds to significant differences (*p < 0.05, **p < 0.01, ***: *p* < 0.001, ****p < 0.0001)

Quantitative PCR assays showed that the expression of *SOX2* and *NANOG* was increased in the overexpression group at the D5 blastocyst stage, but that there was no significant difference in the expression of *CDX2*, *TEAD4*, and *DAB2* (Figure 6A). In the D7 BL stage, the expression of *SOX2* was increased, but there was no significant difference in the expression of *NANOG* (Figure 6B). Interestingly, the expression of *SOX17* was significantly decreased, which was consistent with the decrease in the number of SOX17‐positive cells in the immunofluorescence data. As in the D5 blastocyst stage, there was no difference in the expression of trophoblast‐related genes, and the expression of *CDK4* was significantly increased. These results suggest that OCT4 expression affects other pluripotent‐related gene expressions and second cell fate decisions. However, since OCT4 overexpression did not reduce trophoblast‐associated gene expression in the blastocyst, it suggests that OCT4 overexpression did not lead to ICM fate in all blastomeres.

## 3 DISCUSSION

### 3.1 Species‐specific features of OCT4 during blastocyst formation

In this study, we analyzed the role of OCT4 in lineage specification and proliferation during the development of porcine preimplantation embryos. Many studies have revealed that OCT4 plays an essential role in ICM formation in mammalian embryo development, but species‐specific studies are needed because the expression pattern and role of genes in the blastocyst are different for each species. In the absence of OCT4 in mouse embryos, ICM could not be formed and differentiated into trophoblasts.^9^ In addition, the OCT4 repression of mouse ESCs‐induced differentiation into trophectoderm cells.^41^ On the other hand, one study reported that it was difficult to contribute to the TE lineage when OCT4 target siRNA was injected into porcine embryos.^42^ In this study, the number of trophoblast cells also decreased in OCT4‐targeted embryos, suggesting that OCT4 affects the trophoblast lineage (Table 1). In terms of OCT4 overexpression, more than 50% of OCT4 overexpression in mouse embryos was arrested at the 16‐cell stage.^14^ In contrast, in this study, embryos overexpressing OCT4 developed into blastocysts and exhibited abnormal expressions of SOX2 and SOX17 (Figure 5C). The differences in the results regarding the role of OCT4 between these species can be considered to be related to the location of OCT4 expression. In mouse blastocysts, OCT4 expression is limited to ICM,^43^ but in pigs, OCT4 expression in blastocysts persists for a long time in trophoblasts.^11^ In addition, the developmental arrest caused by the overexpression of OCT4 in mouse embryos resembles that of SOX2 overexpression, which is expressed only in the ICM of previous porcine blastocysts.^18^ It can be inferred that developmental arrest occurred because an asymmetric distribution of ICM‐specific genes failed to occur due to overexpression before the morula stage. Taken together, these results suggest that differences in the spatiotemporal expression patterns of pluripotent genes during mammalian blastocyst formation represent species‐specific features.

### 3.2 OCT4 affects both lineage specifications

In this study, we investigated whether the expression level of OCT4 affects cell fate decisions in pig blastocyst formation stage. In the first cell fate decision, the absence of OCT4 expression prevented ICM formation, leading to the reduced expression of SOX2 (Figure 3). On the other hand, most of the cells overexpressing OCT4 expressed SOX2, and the trophectoderm characteristics were suppressed (Figure 5). Among ICM, cells with highOCT4 staining intensity were differentiated into epiblasts, and cells with low‐intensity OCT4 were differentiated into primitive endoderms. This result is different from mouse embryos. In mouse blastocyst, there was no difference in OCT4 expression between cells expressing high levels of SOX2 and cells expressing high levels of SOX17.^44^ As previous results showed that mouse ESCs in which OCT4 was depleted through RNA interference were mainly included in the extraembryonic endodermal lineage in chimeric embryos,^45^ further comparative studies are needed to determine the differences in OCT4 between the two species. Meanwhile, in the results of this study, the number of SOX17‐positive cells decreased and the number of SOX2‐positive cells increased in D7 blastocysts injected with OCT4 mRNA (Figure 5C), suggesting that OCT4 influences secondary cell fate decisions. Previous studies have shown that threonine 343 phosphorylation of OCT4 plays an important role in determining the second cell fate of mouse embryos.^46^ Accordingly, it can be expected that overexpression of OCT4 breaks the balance between phosphorylated and non‐phosphorylated OCT4, resulting in a biased cell fate decision. Therefore, future studies on the relationship between OCT4 staining intensity and phosphorylation should shed light on the role of OCT4 in second lineage specification.

### 3.3 The effects of OCT4 on embryo cell proliferation

Pluripotent‐related genes and cell proliferation are closely related. In particular, during embryonic development, proliferation is strictly controlled, and the mammalian embryo has a very short division time during preimplantation embryogenesis.^47^, ^48^, ^49^ Thus, OCT4 knockout in human embryos leads to a decrease in the total cell number of the blastocyst.^29^ In addition, the total cell number changes according to the expression level of SOX2 in the pig pre‐implantation blastocyst.^18^ Since the embryo proliferation rate determines embryo quality, it is necessary to investigate the interrelationship between pluripotency and proliferation. Our results suggest that OCT4 affects proliferation during porcine preimplantation embryo development. In this study, it was confirmed that the expression of OCT4 and CDK4 expression are related. CDK4 is a G1‐related gene that is expressed higher in human ES cells than in somatic cells.^50^ In addition, OCT4 is required for cell proliferation in the post‐implantation stage as well as in the pre‐implantation stage in the mouse embryo.^51^ According to the above studies, we speculate that OCT4 influences the G1–S transition of embryonic and embryonic stem cell proliferation.

## 4 CONCLUSION

In conclusion, in the present study, we worked towards clarifying the role of OCT4 during the development of porcine preimplantation embryos through gene knockout and overexpression. OCT4, a core pluripotent gene in preimplantation porcine embryos, determines cell fate according to its expression level. We found that OCT4 knockout in porcine embryos plays a crucial role in ICM formation and cell proliferation. The distinction from mouse embryos was that the expression level of OCT4 was different between epiblast and primitive endoderm in porcine D7 blastocyst. Furthermore, we found that OCT4 affects the second lineage specification through the inhibition of primitive endoderm differentiation in OCT4 overexpressing porcine embryos. However, the precise mechanisms of OCT4 in first and second cell fate decisions and the species‐specific pluripotent gene network remain to be elucidated. Finally, our data provide new insights into the species‐specific characteristics of OCT4 and increases our understanding of the pluripotent network in preimplantation embryo mammalian embryogenesis.

## 5 MATERIALS AND METHODS

The care and experimental use of pigs were approved by the Institute of Laboratory Animal Resources, Seoul National University (SNU‐140328‐2). Unless otherwise stated, all chemicals were obtained from Sigma–Aldrich Corp. (St. Louis, MO, USA).

### 5.1 In vitro embryo production

The ovaries of the prepubertal gilts were obtained from a local slaughterhouse (Anyang‐si, Gyeonggi‐do, Korea) and transferred to the laboratory in warm saline. Cumulus‐oocyte complexes (COCs) were collected by aspirating 3‐ to 7‐mm follicles of the prepubertal gilts using a 10‐ml syringe with an 18‐gauge needle. Sediments were washed with TL–HEPES–PVA medium, and oocytes with compact cumulus cells and granulated cytoplasm were selected for in vitro maturation. The washed COCs were cultured in tissue culture medium (TCM‐199; Life Technologies, Carlsbad, CA, USA) containing 10 ng/ml of epidermal growth factor, 1 mg/ml of insulin, and 10% porcine follicular fluid for 44 h at 39°C in 5% CO_2_ and 100% humidity. The COCs were matured with 10 IU/ml of gonadotropin hormone, pregnant mare serum gonadotropin (Lee Biosolutions, Maryland Heights, MO, USA), and human chorionic gonadotropin for the first 22 h. The COCs were then matured under hormone‐free conditions. To generate parthenotes, cumulus‐free oocytes were activated with an electric pulse (1.0 kV/cm for 60 ms) inactivation medium (280 mM mannitol, 0.01 mM CaCl_2_, 0.05 mM MgCl_2_) using a BTX Electrocell Manipulator (BTX, CA, USA), followed by 4 h of incubation in PZM3 medium containing 2 mmol/L of 6‐dimethylaminopurine.

### 5.2 Production of CRISPR/Cas9 vectors and *OCT4* mRNA

The candidate targeting sequence against the pig *OCT4* gene was selected using the CRISPR gRNA design tool (https://chopchop.cbu.uib.no) to improve gene‐targeting efficiency and minimize off‐targeting effects. DNA oligonucleotides carrying the target sequences were constructed by adding PAM sequences (Table S1). The candidate DNA construct for OCT4 was inserted into the pX330 plasmid and validated using the pCAG‐EGxxFP reporter system.^52^ The OCT4‐pX330 constructs and pCAG‐EG(pig OCT4)FP constructs were introduced into porcine fetal fibroblast (pFF) cells plated in 12‐well plates (300 ng/well) using Lipofectamine 3000 Reagent (Thermo Fisher Scientific, Waltham, MA, USA). EGFP fluorescence was observed under a fluorescence microscope 48 h after transfection. The thermal cycler was used for the target sequencing of the genomic DNA of microinjected single BLs. After destroying cells by repeating high and low temperatures in ultrapure water and single BL, PCR was performed using porcine OCT4 gDNA‐specific primers (Table S1) and 2x PCR master mix solution (iNtRON Biotechnology, Korea). Amplified PCR products were analyzed by an ABI PRISM 3730 DNA Analyzer (Applied Biosystems, Foster, CA, USA). Finally, the selected guide sequence was synthesized sgRNA using the CRISPR/Cas9 service (Comsmo Genetech, Seoul, Korea). Cellular porcine OCT4 mRNA was extracted from porcine embryonic stem cells using TRIzol1 reagent (Invitrogen, MA, USA) according to the manufacturer's instructions. Complementary DNA was synthesized using a high‐capacity RNA‐to cDNA Kit (Applied Biosystems, CA, USA) according to the manufacturer's instructions, producing a final volume of 20 μl. Porcine OCT4 cDNA was cloned using 2x PCR master mix solution (iNtRON Biotechnology) and porcine OCT4 cDNA‐specific primers. Amplified PCR products were subjected to TA cloning and analyzed by an ABI PRISM 3730 DNA Analyzer (Applied Biosystems). Porcine OCT4 mRNA was synthesized and modified (RNA coding sequence, 5′ cap, 3′ poly A tail, and 5′ and 3′ UTR) by Bioneer (Bioneer, Daejeon, South Korea).

### 5.3 Microinjection of RNA into parthenotes

For the pOCT4 CRISPR/Cas9 knockout assay, 1 μl of 20 ng/μl commercial Cas9 mRNA (Thermo Fisher Scientific) and 1 μl of 10 ng/μL sgRNA were added to 8 μl of Media‐199 (Gibco). For the pOCT4 overexpression assay, 1 μl of 250 ng/μL pOCT4 mRNA in 4 μl of Media‐199. One hour after PA, the embryos at the one cell stage were injected with two pL of RNA solution in manipulation media. The microinjection procedure was conducted using a micromanipulator (Eclipse TE2000, Nikon) with a Femtotip II microinjector (Eppendorf). After microinjection, the embryos were washed and then cultured in PZM3 media for 6 days.

### 5.4 Immunocytochemistry

Each stage of embryos without zona pellucida was fixed in 4% paraformaldehyde for 15 min at room temperature. The fixed samples were permeabilized using 1% Triton X‐100 for 1 h at room temperature and then washed three times with phosphate‐buffered saline (PBS). The embryos were blocked using 10% goat serum or donkey serum in PBS for 1 h at room temperature. The samples were stained with anti‐SOX2 (5 μg/ml), anti‐NANOG (1 μg/ml), anti‐OCT4 (1 μg/ml), anti‐SOX17 (1 μg/ml) and anti‐CDX2 (1 μg/ml) antibodies in PBS containing 10% goat serum or donkey serum at 4°C overnight (Table S2). After washing three times in washing solution (PBS with 0.2% Tween‐20 and 1% BSA for 10 min), the embryos were incubated with goat anti‐rabbit Alexa 488 (Invitrogen), anti‐rabbit Alexa 555 (Invitrogen), or donkey anti‐rabbit Alexa647 (Invitrogen) antibodies in PBS with 10% goat serum or donkey serum at RT for 1 h. All samples were washed three times with washing solution after secondary antibody treatment. To immunostain the three antigens together, three primary antibodies were applied to the samples one by one. Immunostained embryos were mounted on a slide glass with Prolong Gold with DAPI (Invitrogen) and cured for more than 24 h. We have described the list of antibodies in Table S2. Images of stained cells were captured using an inverted fluorescence microscope and processed by the ImageJ program. ImageJ was used to merge the fluorescence images and measure the fluroescnece staining intensity.

### 5.5 Confocal imaging process

Confocal immunofluorescence images were taken with a Leica SP8X (Leica Microsystem, Wetzlar, Germany) and processed by the Fiji (ImageJ) program.

### 5.6 Quantitative real‐time polymerase chain reaction

Total RNA from the pooled embryos at each stage of in vitro‐produced embryos (2–3‐cell, *n* = 20; 4‐cell, *n* = 20; 6–8‐cell, *n* = 20; morula, *n* = 10; and blastocysts, *n* = 10) was isolated using an Arcturus® PicoPure® RNA Isolation Kit (Applied Biosystems) following the manufacturer's instructions. cDNA was synthesized using a High‐Capacity RNA‐to‐cDNA Kit (Applied Biosystems). The cDNA samples were amplified using Power SYBR Green Master Mix (Applied Biosystems) containing 1 pmol of each primer set that is listed in Table S3 in a 10 μl reaction volume. Amplification and detection were conducted using the ABI 7300 Real‐Time PCR System (Applied Biosystems) under the following conditions: one cycle of 50°C for 2 min and 95°C for 10 min, followed by 40 cycles of denaturation at 95°C for 15 s and annealing/extension for 1 min (annealing/extension temperatures were dependent on each primer set). The dissociation curves were analyzed, and the amplified products were loaded onto gels to confirm the specificity of the PCR products. The relative expression level was calculated by normalizing the threshold cycle (Ct) values of each gene to that of the reference gene beta‐actin (ACTB) via the delta–delta Ct method.

### 5.7 Statistical analysis

Statistical analysis of the data was performed using GraphPad Prism Software (version 7; San Diego, CA, USA). Significant differences in gene expression among the experimental groups were determined by one‐way analysis of variance followed by Tukey's multiple comparison test. Differences were considered significant at *p* < 0.05 (*p < 0.05 and **p < 0.01, ***p < 0.001, ****p < 0.0001 in the figures). Data are presented as the mean ± standard error of the mean (SEM).

## AUTHORS CONTRIBUTIONS

Mingyun Lee, Jong‐Nam Oh, Seung‐Hun Kim, Kwang‐Hwan Choi, Dong‐Kyung Lee and Chang‐Kyu Lee designed research; Mingyun Lee, Jong‐Nam Oh, Seung‐Hun Kim, Gyung Cheol Choe and Jinsol Jeong performed research; Mingyun Lee, Jong‐Nam Oh, Seung‐Hun Kim, Kwang‐Hwan Choi, Dong‐Kyung Lee analyzed data; Mingyun Lee and Chang‐Kyu Lee wrote the paper; Chang‐Kyu Lee finally approved the manuscript.

## CONFLICT OF INTEREST

The authors declare no conflicts of interest.

## Supporting information


**Table S1** Primers used in this study.
**Table S2** List of antibodies.
**Table S3** Oligonucleotide sequences used in quantitative PCR.

## Data Availability

The datasets used in the current study are available from the corresponding author on reasonable request by email.
