# Supplementary material for: Changes in OCT4 expression play a crucial role in the lineage specification and proliferation of preimplantation porcine blastocysts
Source: Cell Prolif. 2022 Jul 26;55(11):e13313. doi: 10.1111/cpr.13313 (PMC9628253; doi:10.1111/cpr.13313)
Supplement: Supplementary file 1 — Table S1 Primers used in this study. Table S2 List of antibodies. Table S3 Oligonucleotide sequences used in quantitative PCR. [file CPR-55-e13313-s001.docx]

**Table S1 Primers used in this study.**

| Primers | Sequence (5’→3’) | Description |
| --- | --- | --- |
| pCAG-pOCT4-1 | F: tgaggatccgctagcctgcagacacctggcttccgacttc | pCAG-EG(porcine OCT4)FP construction |
|  | R: atcgaattcgtcgacctgcattctccttgtccagcttcgc |  |
| pCAG-pOCT4-2 | F: tgaggatccgctagcctgcaggaaggtgttcagccaaacg |  |
|  | R: atcgaattcgtcgacctgcagagacccagcagcctcaaaa |  |
| OCT4 gRNA-1 | F: CACCGccgggctgggttgatcctcggac | gRNA-1 on-target site |
|  | R: CGGCCCGACCCAACTAGGAGCCTGCAAA |  |
| OCT4 gRNA-2 | F: CACCGcaacaacgagaatctgcaggag | gRNA-2 on-target site |
|  | R: CGTTGTTGCTCTTAGACGTCCTCCAAA |  |
| OCT4 gRNA-3 | F: CACCGtcaagaacatgtgtaagctgcgg | gRNA-3 on-target site |
|  | R: CAGTTCTTGTACACATTCGACGCCAAAC |  |

Underlined nucleotides refer to gene-specific regions, and lowercase letters indicate overhangs.

**Table S2 List of antibodies.**

| Primary Antibodies | Target | Host | Company | Catalog Number |
| --- | --- | --- | --- | --- |
|  | SOX2 | Rabbit | Millipore | AB5603 |
|  | OCT4 | Rabbit | Santa Cruz | sc-9081 |
|  | NANOG | Rabbit | Peprotech | 500-P236 |
|  | SOX17 | Goat | R&D systems | AF1924 |
|  | CDX2 | Rabbit | Abcam | AB7654 |
| Secondary Antibodies | Fluorescent dye | Target/Host | Company | Catalog Number |
|  | Alexa594 | Rabbit/Goat | Invitrogen | A-11012 |
|  | Alexa594 | Rabbit/Donkey | Invitrogen | A-21207 |
|  | Alexa488 | Rabbit/Chicken | Invitrogen | A-21441 |
|  | Alexa488 | Goat/Donkey | Invitrogen | A-11055 |
|  | Alexa555 | Goat/Donkey | Invitrogen | A-21447 |
|  | Alexa555 | Rabbit/Goat | Invitrogen | A-21428 |
|  | Alexa647 | Goat/Donkey | Invitrogen | A-32849 |

**Table S3 Oligonucleotide sequences used in quantitative PCR.**

| Primer | Sequence (5’→3’) | Tm | Size |
| --- | --- | --- | --- |
| *OCT4* | F: GCTGGAGCCGAACCCCGAGG | 68°C | 150 |
|  | R: CACCTTCCCAAAGAGAACCCCCAAA |  |  |
| *SOX2* | F: CGGCGGTGGCAACTCTAC ' | 64°C | 100 |
|  | R: TCGGGACCACACCATGAAAG |  |  |
| *NANOG* | F: CATCTGCTGAGACCCTCGAC | 60°C | 195 |
|  | R: GGGTCTGCGAGAACACAGTT - |  |  |
| *SOX17* | F: GCAAGATGCTGGGCAAGT | 60°C | 112 |
|  | R: TTGTAGTTGGGGTGGTCCTG |  |  |
| *CDX2* | F: CAGCGGCGGAACCTGTG | 63°C | 92 |
|  | R: ACTCGGTATTTGTCTTTCGTCCTG |  |  |
| *TEAD4* | F: AAGGCCGGCACCATTACCT | 60°C | 231 |
|  | R: CAGCTCATTCCGACCGTACAT |  |  |
| *DAB2* | F: TGGGAGTGAGGCCCTAATGA | 58 | 111 |
|  | R: GGACTACTTAGGTCGGGAGGT |  |  |
| *CDK4* | F: 5′-GCATCCCAATGTTGTCCG | 60°C | 126 |
|  | R: 5′-GGGGTGCCTTGTCCAGATA |  |  |
| *ACTB* | F: GTGGACATCAGGAAGGACCTCTA | 60°C | 131 |
|  | R: ATGATCTTGATCTTCATGGTGCT |  |  |
